# Emotional Intelligence and Cognitive Flexibility are Key Influences in Enabling Staff to Uphold Human Rights Values for People Living With Dementia in Care Homes

**DOI:** 10.1177/14713012251392304

**Published:** 2025-11-20

**Authors:** Lesley Butcher, Sofia Vougioukalou, Teena Clouston

**Affiliations:** 1 Adult Nursing, School of Healthcare Sciences, 2112 Cardiff University, Cardiff, UK; 2 School of Medicine, 2112 Cardiff University, Cardiff, UK; 3 Occupational Therapy, School of Healthcare Sciences, 2112 Cardiff University, Cardiff, UK

**Keywords:** dementia, care homes, human rights, fairness, respect, equality, dignity, autonomy, emotional intelligence, cognitive flexibility, care home ethos, organisational culture

## Abstract

**Background:** People with dementia experience discrimination and treatment that contravenes their human rights in health and social care settings. Human Rights law is complex, and real-world application can be confusing. Researchers used the FREDA (Fairness, Respect, Equality, Dignity, Autonomy) framework to provide context to focus group discussions that explored the research question: ‘What are the barriers and facilitators to upholding human rights for people living with dementia in care homes?’. **Method:** Seven focus groups of 90 minutes were conducted. Vignettes and open questions guided discussion. Groups consisted of 7-9 people of heterogenous backgrounds including care home staff (N = 35 from 20 care homes), people with dementia (N = 5), family members of someone with dementia (N = 5), and student nurses (N = 7). Thematic analysis was completed using the Braun and Clarke (2006) inaugural framework with the 6-phase analytical process and reflexive approach. **Results:** Three overall themes were elicited through this study, including: *1*. *Personal/individual attributes including subthemes: emotional intelligence, cognitive flexibility and education & experience*. *2*. *Organisational culture including subthemes: care home ethos, organisational flexibility, and staff morale*. *3*. *External influences including subthemes: family and visitors, and social care funding and support*. The largest barrier to supporting human rights principles reflected *care home ethos*, followed by *organisational flexibility*. The greatest facilitator was *emotional intelligence*, then *cognitive flexibility.* **Conclusions:** This study responds to some of the recommendations from previous research that considered Human Rights approaches to people living with dementia in care homes. It addresses the specific suggestion to identify staff attitudes and understandings that might translate to meaningful enhancements in care relating to FREDA principles of human rights. This is the first study to determine that emotional intelligence and cognitive flexibility are key influences in enabling care home staff to uphold values of human rights for people living with dementia in homes.

## Introduction

People with dementia experience social inequalities, injustices and discrimination that contravene their human rights (Alzheimer’s Society, 2015; Steele et al., 2023). There is known social stigma to the condition (Nguyen & Li, 2020), and health professionals contribute to this (Digby et al., 2017; Gove et al., 2016). Health and social care settings present a heightened risk of exposure to human rights abuses for people with dementia (Dixon et al., 2022) which may include coercive or forced treatment or procedures (World Health Organisation [WHO] 2017b). A recent systematic review (Liu et al., 2024) revealed that almost half (45.2%) of community-dwelling people living with dementia were subjected to involuntary treatment that included non-consensual care, use of psychotropic medication and physical restraint. Despite regulations being in place to support freedom and choice, there is still significant use of physical and chemical restraints within care homes and acute hospital settings (WHO, 2023). Violations of human rights are associated with poor health outcomes (WHO, 2017b) which is incongruous with the purpose of health and social care to promote health and wellbeing (Department of health, 2022).

### Care Home Context

Approximately 70% of care home residents are living with dementia (Alzheimer’s Society, 2016). There is evidence of wide disparity among care home services (Killett et al., 2016). Inspection reports revealed that 34% of care homes demonstrated variable or poor care in relation to people’s mental health, emotional care, and social needs (Care Quality Commission [CQC] 2021). People with dementia can experience dehumanisation, social isolation, and inequality (Steele et al., 2023) in care home settings.

The pressure associated with working in care homes is well researched (Islam et al., 2017; Pelissier et al., 2015) and related to several key factors, such as homes experiencing a high staff turnover (Backman et al., 2024). A major influence of this is poor job satisfaction and burnout (Isaksson, 2013) which is said to be higher than those who work in other settings such as hospitals (White et al., 2020). Care home nurses are reported to have poor mental health associated with a stressful work environment (Zhang et al., 2016). Certain practices within the role can negatively affect the dignity and respect of residents and care workers alike (Johnson, 2015, 2023). Care home staff experience anxiety and frustration that can lead to depersonalisation, controlling behaviour and avoidance of the person with dementia (Beeber et al., 2014; McKenzie et al., 2016). Moreover, caring for people with dementia in care homes is often complex and unpredictable, increasing levels of stress (Feast et al., 2016; Keenan et al., 2020).

### A Human-Rights Based Approach

A Human-Rights based approach is advocated in health and social care settings (WHO, 2017a). The Human Rights Act (1998) integrates most of the rights protected under the European Convention on Human Rights (ECHR). Human rights are based on core principles such as fairness, respect, equality and dignity (British institute of Human Rights, 2016). The expectation to deliver person-centred care is well documented (Byrne et al., 2020; Kitwood, 1997; Kitwood & Brooker, 2019; Rooney, 2019). A human rights-based approach adds fortitude and a legal framework to principles of person-centred care (Butchard & Greenhill, 2015), emphasising there is a legal duty under the Human Rights Act (1998) to uphold said principles.

### The FREDA Framework

There are barriers to understanding the legal composition of the human rights act and its practical translation to the care setting (Fitzgerald et al., 2020). The FREDA framework (Curtice & Exworthy, 2010) highlights values of Fairness, Respect, Equality, Dignity and Autonomy. Although not legally binding, FREDA is an internationally recognised acronym for the principles outlined in the Human Rights Act (1998) legislation and is a means through which human rights can be interpreted (Health Information and Quality Authority [HIQA] 2019). This approach allows healthcare practitioners to apply essential legal principles of human rights legislation within a practical context. FREDA has also been adopted by national regulators of health and social care in their approach to care home inspections (Care Inspectorate Wales [CIW], 2023, CQC, 2024) to ensure that human rights-based care is delivered, and has been used as a guide to develop national standards for health and social care (HIQA 2019).

This research explores the experiences of key stakeholders to ascertain the barriers and facilitators to upholding the following key FREDA principles.

*Fairness* considers the opinions of people, ensuring their viewpoints are heard and taken seriously (Curtice & Exworthy, 2010). This principle maintains that the person should be at the centre of any decision made about them (McGrane et al., 2024) and that they are treated without discrimination.

*Respect* refers to honouring the rights, standards, and philosophies of a person (HIQA 2019) including their cultural identity (Freeman, 2022). *Equality* concerns the allowance of equal opportunities for involvement regardless of individual characteristics, ensuring nobody is disadvantaged (James et al., 2022). *Dignity* is a multifaceted concept which encompasses a sense of self-acknowledgment, respect and autonomy (Banerjee et al., 2021). It is experienced as being valued as a unique individual (Lindwall 2021) and is closely linked to privacy and communication (Ekpenyong et al., 2021). *Autonomy* is about enabling people to maintain control over their lives and to make choices that reflect their unique personality (Kaplan & Bentwich, 2023). When living in a care home, a person’s sense of autonomy and control are factors that contribute to their sense of feeling ‘at home’ (Rijnaard et al., 2016).

Kinderman et al. (2018) applied a human-rights based approach to people living with dementia in hospital and care home settings. Findings demonstrated that although the knowledge and attitudes of staff improved, there was no enhancement in the quality of care delivered, nor did it improve wellbeing for people with dementia. It was recommended that future research should focus on understanding the barriers to translating attitudinal changes into behaviour. This research therefore aimed to uncover some of the wider contributors to what impedes staff from caring for people with dementia in a way that upholds their human-rights principles.

By exploring the experiences and understandings of key stakeholders, this qualitative study aimed to identify the barriers and facilitators to upholding principles of human rights for people living with dementia in care homes. The FREDA values (Curtice & Exworthy, 2010) were used as a validated framework to assist the researchers in analysing participant responses to identify aspects of fairness, respect, equality, dignity and autonomy. In gauging participant responses, researchers aimed to establish the conditions that might impede someone with dementia from being cared for in a way that supported their human rights. Equally, it was important to ascertain the approaches and practices that promoted and enhanced care in a way that is consistent with upholding human rights values.

## Design and Methods

### Research Approach

This research was ethically approved by the Research Ethics Committee for the School of Healthcare Sciences, Cardiff University, prior to advertisement and recruitment. Focus groups were considered the most suitable method to elicit a range of opinions and experiences from participants. Acknowledging that the interaction between participants allows for a rich and potentially more detailed discussion (Tümen and Ahmed 2021), the collected data could then be compared with other groups to establish patterns of shared perspectives (Rogo, 2024). While homogenous sampling can be beneficial to research validity (Femdal & Solbjør, 2018), researchers chose a heterogenous group for this study. The purpose was to create a flow of discourse from people from different backgrounds to elicit a broader range of perspectives and potentially offer a more complete representation (Boswell & Cannon, 2019) of the situation in care homes.

### Participant Recruitment

Purposive sampling (Jacobsen, 2020) was used to recruit participants from mixed backgrounds, who could offer different viewpoints in answering the research question. Participants included care home staff (N = 35) from 20 care homes across South Wales, people living with dementia (N = 5), family members of people living with dementia (N = 5), and student nurses who had a recent care home placement (N = 7).

Care home staff responded to invitation letters, emails and social media advertisements. Inclusion criteria comprised any member of staff who provided care and treatment for those living with dementia in care homes. This included nurses, managers, care assistants and activity coordinators. Inclusion criteria for people living with dementia were those who had a diagnosis of dementia and the capacity to consent to the study. This was ascertained prior to recruitment. Most of the participants living with dementia (N = 4) were known to the lead researcher through previous teaching collaborations. They were contacted via email and provided with a participant information sheet and consent form to review approximately two months in advance of the research to allow time to consider their involvement and for the opportunity to ask questions to clarify the information. The fifth participant with dementia expressed an interest in participating based on an invitation letter sent to the manager of his care home in which he resided. The care home manager ascertained that he had the capacity to consent to the study. Acknowledging that consent is a continuous process and should be re-established at each encounter (Quinn et al., 2024), capacity to consent was re-checked by the researcher on the day of participation. As with all participants, informed signed consent was obtained. Family members included those whose loved one either currently resided in a care home or did so until their end of life. They were recruited via social media advertisement. Further information was clarified via email prior to agreement to participate. Inclusion criteria were not restricted to those who were ‘family’ through birth or domestic partnership but could have included anyone considered to be a ‘significant other’ to the person with dementia. However, only close family members responded to the study invitation. Student nurses were recruited via an internal ‘expression of interest’ advertised within Cardiff University’s undergraduate nursing programme. Inclusion criteria comprised student nurses who had a recent care home placement experience. Student nurse participants were not previously known, as individuals, to the researchers prior to the study.

Care home staff were in a key position to respond to the research question. Likewise, inclusion of people with dementia was essential (NIHR, 2020). As people living with the condition, their inclusion can improve the quality and validity of the research and recognises their right to be involved (McKeown, 2019). This reflects the values of equality and choice, which signifies the essence of this research. Student nurses have made a valuable contribution to previous research (Edwards et al., 2017; Jones, 2017; Saville, 2018). While they are clinically inexperienced, they offer a fresh perspective on clinical practice. They are ideally placed to identify the theory–practice gap (Palese et al., 2019).

### Focus Groups

Seven focus groups were conducted within the greater context of a civic mission project aimed to engage external partners and improve empathy for people living with dementia. These discussions took place on 15^th^ November 2019 simultaneously for 90-min. Group facilitators were from academic and professional backgrounds, mostly from Cardiff University. Groups consisted of 7-9 people of mixed backgrounds (Table 1). Each 90-min discussion was digitally recorded and professionally transcribed verbatim. Group discussions were all conducted in the English language. Vignettes (N = 5) (Table 2) were used to generate initial discussion. These were based on the lead researcher’s lived experiences in practice. Scenarios were chosen for their potential to generate discussion on human rights values. Vignettes were presented to the research team and focus group facilitators for feedback on their appropriateness prior to inclusion. Group facilitators were provided with some standard open questions to guide discussion if needed (Table 2). Otherwise, there were no other structured questions included, to allow for a more natural flow to the dialogue and standardisation across all 7 groups.

**Table 1.** Summary of Participants in Each of the 7 Focus Groups, and Coded Comments in Relation to Subthemes and Themes

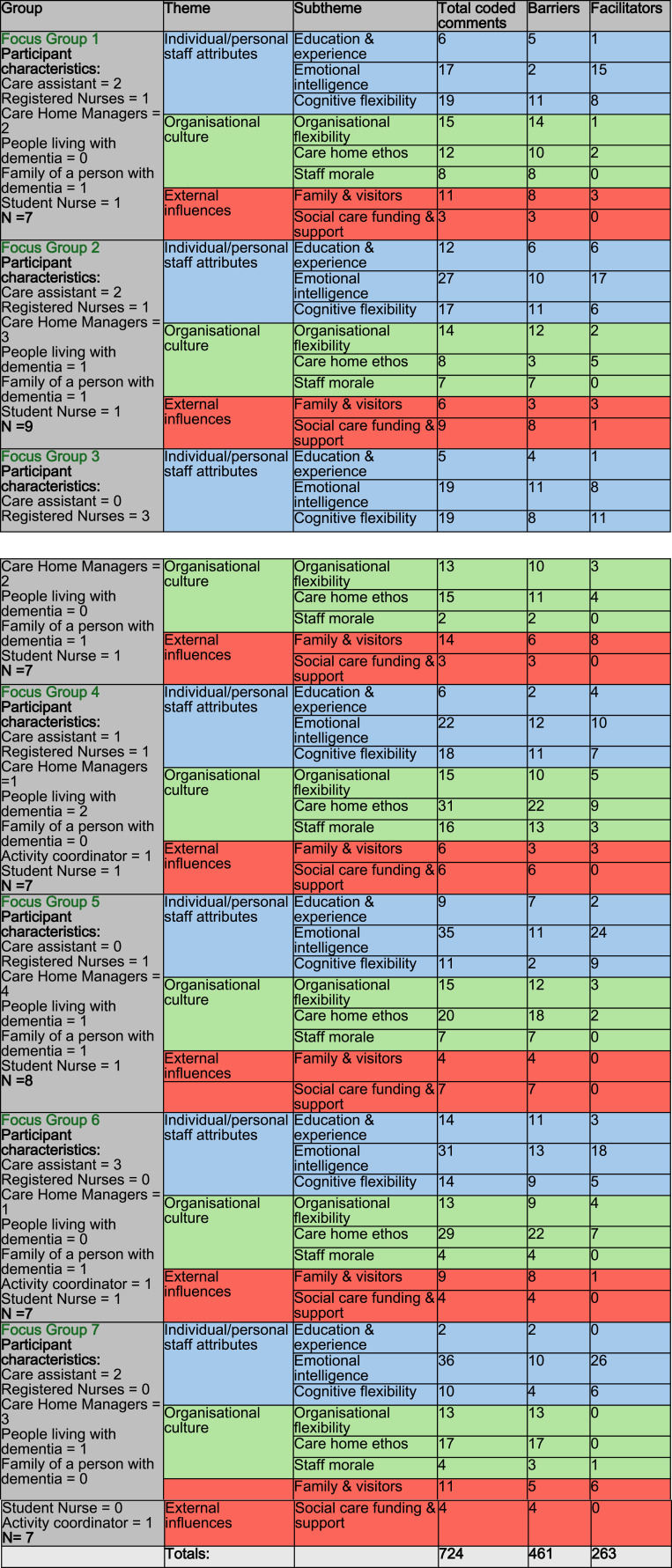

**Table 2.** Vignette Example

Vignette 1: (Fairness, dignity, respect, autonomy)
Bill is living with dementia in a care home. He can walk with a Zimmer frame; however he is unsteady on his feet and needs staff supervision when walking. Bill tries to get up during a mealtime. Staff are busy assisting residents with their food. A member of staff calls out from across the room:
“Bill- sit down lovely!”
Bill says he wants to use the toilet. He stands up and reaches for his frame. Another nearby staff member moves the frame out of his reach and assists him to sit back down. The first staff member shouts across the room:
“Bill- you’ve got a pad on- sit back down now please”
1. What are your initial thoughts on this?
2. Why do you think the staff member did not assist Bill to the toilet?
3. How might these reasons mentioned be overcome?

Field notes were taken by the focus group facilitators during the discussion. These consisted of facilitators noting phrases that had appeared to be relevant to the research question. Participants were allocated to groups in advance to ensure an even distribution of backgrounds and representation in each group. Participants from the same care home were assigned to different groups to limit any possible influence of pre-existing dynamics or power differentials (Sim & Waterfield, 2019). Two groups did not have a person with dementia or carer represented due to unavailability. All participants were provided with an information sheet and signed a consent form prior to taking part.

### Thematic Analysis

The data were analysed using Braun and Clarke’s (2006) inaugural method of thematic analysis using the 6-phase analytical process, then refined with use of the same authors’ more contemporary clarifications on their initial methodology (Braun & Clarke, 2019, 2024a; 2024b, Braun et al., 2022) with emphasis on the process of ‘reflexive’ thematic analysis. A comprehensive summary of the process can be viewed in Table 3.

**Table 3.** Reflexive Thematic Analysis Based on Braun and Clarke (2006) and Braun and Clarke (2024b)

The 6-phase process	Actions taken in each stage	Commentary on the approach
1. Data familiarisation	LB and TC repeatedly read the transcripts separately. Each made observations and noted these as comments within transcribed documents. The audio recordings were replayed to identify additional context (Byrne, 2022). Comments were shared between the two researchers.	LB & TC phrased their comments and coding with different choices of words, but the concepts and shared meanings were comparable among the two researchers.
2. Coding	Codes were generated in the form of concise interpretive statements that were relevant to addressing the research question (specifically the FREDA principles). Several rounds of coding were completed. TC coded 3 and LB coded 4 of the focus group transcriptions.	LB and SV had both been involved in data collection as group facilitators, but TC was not. This provided some objectivity to the analysis.
3. Generating initial themes	LB was mostly responsible for re-examining and refining the data. All transcripts, recordings and coding were shared on a database made accessible to researchers LB, TC and SV, who were able to review, agree, suggest alternative opinions or re-working of themes. Certain codes were recognised as familiar, predominant throughout the data set, and analogous with all 7 focus groups. These were then refined and grouped into ‘patterns of shared meaning’ which Braun and Clarke (2024a, 2024b) define as ‘themes’.	Despite comparable shared meanings concerning the coded data between researchers, Braun & Clarke (2024b) method does not rely on multiple independent researchers’ agreement on codes. They contend that reliance on inter-coder reliability does not guarantee ‘accurate’ coding. In this reflexive approach, the coding process reflects a developing understanding of the data through immersion, and how the researcher construes this through their own interpretive lens.
4. Developing and reviewing themes	On further immersion with, and scrutiny of the data, it was recognised that some initial themes had similarities and were part of a larger subject domain. Hence these became ‘subthemes’ to the larger themes they represented. These were re-checked across the data set to determine relevant representation of the data and the research question.	The data was analysed as a fluid and flexible process of data immersion, reflecting the Braun and Clarke (2019, 2024b) method by deeply engaging, scrutinising and exploring the data. This reinforces the method of ‘recursive and reflective’ analysis. Braun and Clarke (2024b) emphasise that although this is a progressive flow of analysis that develops sequentially from previous phases, it is likely the researcher will need to go back intermittently and re-examine earlier stages, to further refine the themes.
5. Refining, redefining and naming themes	A thematic ‘mind map’ was produced (Figure 1). The codes were re-examined repeatedly against the dataset and the themes. These were separated into codes which clearly reflected *barriers* to upholding FREDA principles, and those that reflected *facilitators*. This allowed the researchers to more specifically ascertain participant understandings of what either helped or hindered a human rights-based approach to caring for people with dementia.	While some subthemes were easily defined, others were indistinct and required further clarification. Subthemes ‘emotional intelligence’ and ‘cognitive flexibility’ were initially one subtheme under the heading ‘emotional intelligence’. However, through re-examination of the data it was established that, although they complement one other, they are in fact two distinct approaches.
6. Writing up	There were numerous comments from participants that highlighted the key elements of each theme. The researchers chose a few of the most applicable participant quotes that reflected elements of the themes and subthemes to include in the manuscript.	Phases 4 and 5 provide examples of what Braun and Clarke describe in their reflexive approach. They note that upon re-examining the data, themes can then be split, merged, or abandoned (Braun & Clarke, 2024b).

### Researcher Reflexivity

Listening to the audio recordings as well as reading transcripts was essential. Some professionally transcribed comments were originally attributed to participants, then later identified by researchers as the voice of a group facilitator. Two facilitators with dementia expertise had inadvertently shared some of their own professional experiences and opinions. On recognising this, researchers excluded those comments from the coding to ensure that only the participants’ comments were included in theme formation, to reduce the potential for researcher bias (Stahl & King, 2020).

It is also noteworthy that four of the participants living with dementia were known to the lead researcher through previous teaching collaborations. Potential bias was mitigated during analysis by ascertaining that all focus group facilitators shared the same vignettes and open-ended questions. The data were scrutinized for any potential ‘leading’ questions directed towards any group member. Personal opinions provided by the facilitators were excluded from analysis. All opinions shared by participants with dementia were their own.

However, the researchers are mindful that opinions provided by facilitators could still have led their group towards what was thought to be expected or ‘acceptable’ statements, hence raising the potential for social desirability bias (Bergen & Labonté, 2020). This is especially the case as the research was held within the context of a civic mission stakeholder event which advocated for the improvement of empathy in people who live with dementia.

## Findings

**Figure 1.**
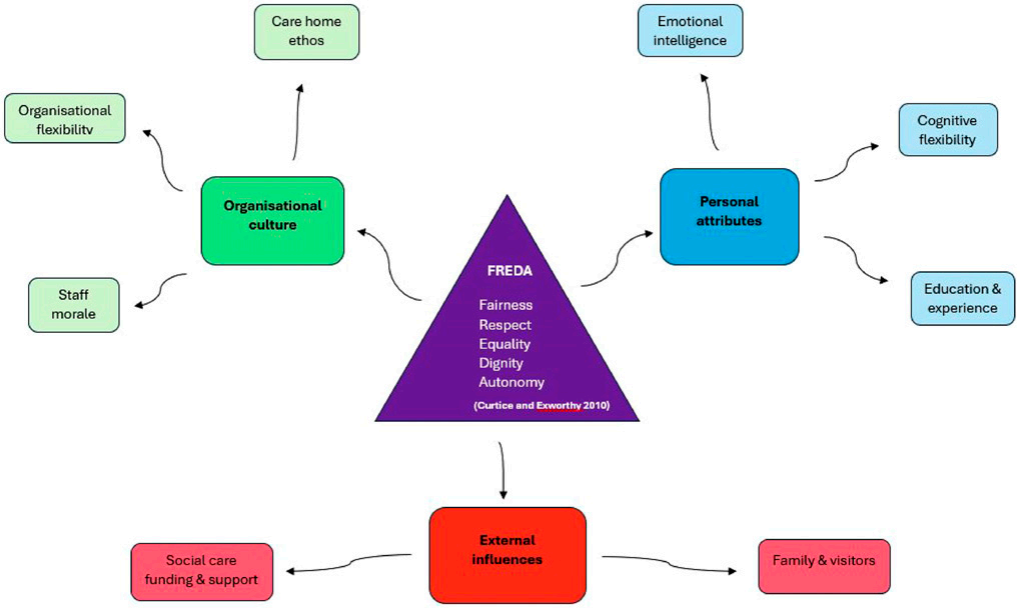
Thematic Mind-Map of Themes and Subthemes

A total of 724 coded comments relating to FREDA principles of human rights were identified. Following analysis, three key themes were established; **Personal/individual staff attributes,** which accounted for 48% of the coded comments**, Organisational culture,** reflecting 38% and **External influences** which represented the remaining 13%. Smaller subthemes represented specific elements of each theme. These are exhibited in Figure 1.

**Figure 2.**
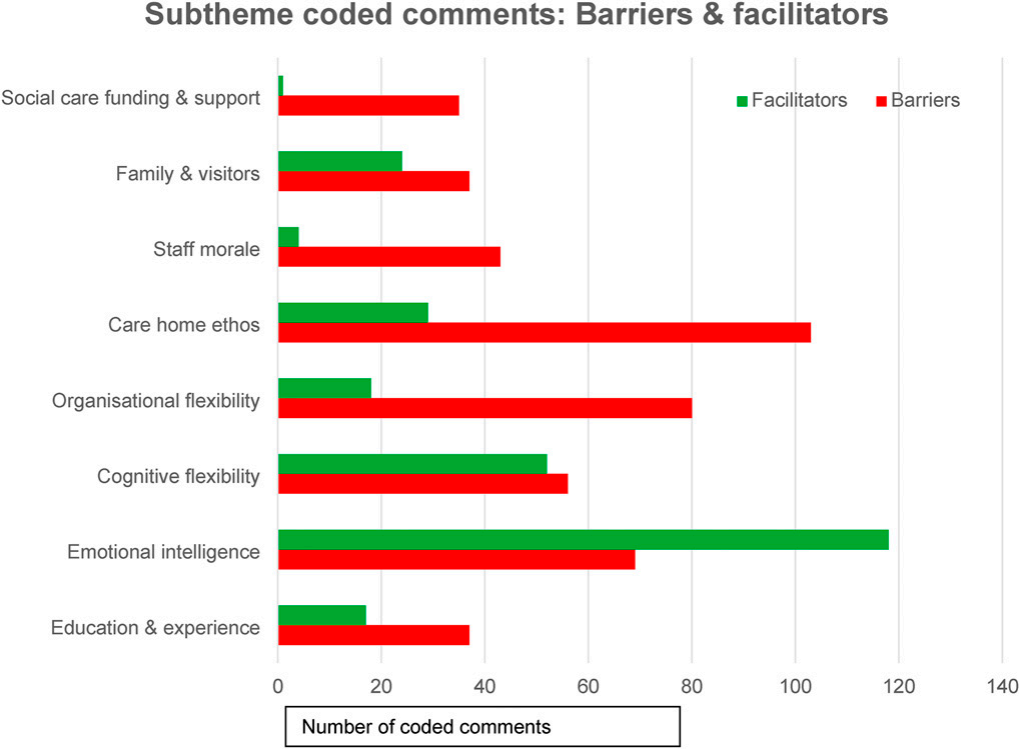
Subtheme Representation of Barriers and Facilitators

Coded comments reflecting each subtheme were categorised into *barriers* and *facilitators* (Figure 2). The most frequently mentioned barrier to upholding human rights principles was *care home ethos*, followed closely by *organisational flexibility.* These are both aspects of the theme ***organisational culture***. Conversely, the greatest perceived facilitator to supporting human rights principles for people with dementia was *emotional intelligence*, followed by *cognitive flexibility*; both are subthemes to the theme ***personal/individual attributes***.

## Theme 1: Personal/Individual Staff Attributes

This theme represents personal qualities reflected by the individual. In this context we refer to staff caring for people living with dementia within the care home who bring their own personal traits, abilities, values and beliefs to the role. This theme encompassed the subthemes **Emotional intelligence, Cognitive flexibility, and Education and experience.**

### Subtheme 1.1: Emotional Intelligence

Emotional intelligence accounted for the highest number of coded comments concerning facilitators (N = 118) and the third highest among the coded comments that reflected barriers (N = 69). Contributing to this subtheme were comments alluding to compassion, empathy, understanding, sensitive communication, and navigating emotions. Participants in every group remarked that it was essential for staff to have compassion. Possessing a benevolent nature was not surprisingly considered to be a facilitator to supporting principles of human rights for any person. Acknowledging the emotional aspects of working with people is highlighted (Table 4), in addition to accepting ones’ own feelings, imperfections and humanity.

**Table 4.** Participant Comments Reflecting Emotional Intelligence

We’re working with the emotions of people, aren’t we? (care home staff)
They don’t need to know [remember] your name, they just need to trust you (care home staff)
We respect that at all times we are just guests here. We may work here on a day-to-day basis, but we are the guests (Care home manager)
It’s about making sure that person is allowed to be the person they are (Person with dementia)
There’s nothing wrong with showing emotion is there? (care home staff)
But we do get things wrong… and we’re human, we’re supposed to get things wrong or how are we going to get things right… (care home staff)

An interesting debate in relation to emotional intelligence emerged from one of the vignettes that raised the question of whether it is acceptable to lie to a person with dementia. The concept of ‘lying’ to a recipient of care denotes loss of trust and relates to FREDA principles of ‘dignity’ and ‘respect’. To consider that it is appropriate to lie to someone with dementia, while choosing to tell the truth to others, infringes on ‘equality’ as a human rights value.

Responses to this depended on experience, background and moral framework. Discussions frequently considered two opposing options: reality orientation or telling the ‘therapeutic lie’. Participants raised the commonly experienced situation of a person with dementia believing incorrectly that their loved one is still alive and is asking for them. While some participants expressed that it was unethical to lie, others contended that the harshness of reality orientation was justification to default to a remedial untruth. One participant with dementia agreed that truth telling in some instances could be distressing, while another was not in favour of lying in general. Some participants used distraction techniques that did not validate the person or acknowledge their underlying need. Others referred to ‘that middle bit’; recognising the gradient of responses available as opposed to concrete perspectives. Some participants referred to evidence-based tools to guide their communication on this subject (Table 5).

**Table 5.** Facilitated Question: “Is it Ok to Lie to Someone With Dementia?”

Yes	Yeah (Care home staff)
	Yes, because you follow the framework (Care home manager)
	There are occasions when you would have to distract or even tell a lie (Person with dementia)
	I wouldn’t say, well, you know, “Mr Davis isn’t here, he’s passed away, Mrs Davis”. Oh, you wouldn’t say that, you just go with the story, I do… (Care Home staff)
No	I don’t agree with that at all (Care home staff)
	It’s not good to lie to people (Person with dementia)
	P1: I was a conference last week… and they called it ‘misdirection’
	P2: It’s called lying isn’t it, let’s not give it a posh term (Care home staff)
Exploring reasons for using the ‘therapeutic lie’	A friend went into hospital, and she died a few weeks ago. And she kept asking for her husband and the children kept saying “Look mum, dad died!” And then she would be upset (Person with dementia)
	People with dementia who are constantly saying “Where’s mum” or “where’s dad” and if you say to them “well how old are you? Oh I’m 98. Well mum’s dead.” They cry and they’re upset because they are grieving because they’ve just lost their parent… (Care home staff)
	I can’t devastate this lady 10 times a day when she asks me. So, our little [white lie] in her best interests is, “He’s on a business trip”. And then she’ll tell you. “Well, when’s he back?” And we’ll say, “Oh, in a couple of days”. (Care Home staff)
Use of redirection	I tend to just ask them about their family and say, “When is John coming in?”- it resets them back to the now… (Care home staff)
Using tools to support decision-making	We embed the [dementia truth inquiry report] framework from the Mental Health Foundation so we would spend time with individuals and assess whether or not it would be suitable for their well-being to tell a lie, or to tell the truth or if there’s that middle bit… (Care Home Manager)
Using validation and understanding	It’s about validating their feelings and listening to what they’re saying… it’s that middle bit between truth and lie… (Care Home Manager)

Another example reflecting the emotional intelligence subtheme was when discussing the use of dignified terminology. Using terms of endearment such as ‘lovely’ or ‘sweetie’ in place of a person’s preferred name can contravene principles of dignity and respect. Some participants held the view that substituted terminology was simply reflective of culture and is therefore acceptable. Others considered that it reflected the individual personality of the staff member, suggesting this would not change. However, most were able to consider that terms of endearment should reflect the way the individual wished to be addressed (Table 6).

**Table 6.** Participant Perspectives on Terms of Endearment

It’s cultural	I come from a small mining village and that’s how everybody speaks to everybody… (Care home staff)
It’s my personality	I do find myself talking like that sometimes, even though that may not be acceptable, but it’s who I am… (Care home staff)
We all have our preferences	P1: It could be patronising
	P2: We don’t know, do we? That might be a way that he likes to be addressed…
	P3: Hmm, and it might not (Care Home staff)
	The one I don’t like is ‘my friend’…’how are you my friend’ and you think….. you know, I’ve never met you before… (Person with dementia)

There were varying levels of emotional navigation exhibited in these examples. Some participants were able to consider the feelings of the person with dementia, and how a certain response might impact on their sense of dignity and respect.

### Subtheme 1.2: Education and Experience

Participants discussed the impact of education and training on their ability to care for people with dementia, particularly in relation to provision of education on communication, compassion and empathy which relate to all aspects of a person’s ability to fulfil FREDA principles within their role. Comparison of barriers to facilitators were N = 37 barriers as opposed to N = 17 facilitators in this subtheme. One topic of repeated discussion was about newly recruited staff lacking experience and the compassion required for the role. Participants described inadequate training, lack of direct supervision and support. Online training was described by some as a ‘tick box’ exercise. Others highlighted a lack of specialist knowledge regarding the educators, with one main trainer allocated to deliver all online training sessions regardless of subject. Concern was raised regarding Further Education (FE) system and whether the certificates of qualification issued reflected the level of training required for the role.

The *education* subtheme crossed over marginally with *emotional intelligence*, in that there was a need for staff to be compassionate and empathetic, but this was not always a priority for training. Regardless, some participants put forth the opinion that compassion and empathy cannot be taught. Others presented more positive experiences of their education provision. These included employing specialist dementia educators to support staff, or engaging specialist training providers with credibility in dementia care, to deliver training (Table 7)

**Table 7.** Perspectives on Education and Training

Inexperienced & unsuitable applicants	Not everybody comes into care work from another care background it might be a change, or it might be the only job available, and they come in and they don’t really understand what is in front of them (Care Home staff)
	Some staff have come into care thinking it’s an easy job. (Care Home staff)
	I would rather take any person who is caring and compassionate and had a real drive for the role than someone who has got all the education but still they’re not a good fit because they are still not able to give the proper care with compassion. I can’t teach that. (Care home manager)
	Sometimes you don’t get the applicants and you kind of feel a bit forced to accept what’s coming through the door (Care Home Manager)
Inadequate training & supervision within the role	There should be supervision as well; somebody to monitor… they come in for personal care and they just go “oh I don’t know how to change her” and there was nobody there to show them. They didn’t have any proper care…they just have to carry on don’t they (Care Home staff)
	It’s like learning next to Nelly… unless you know right and wrong you just copy Nelly and think that that’s right don’t you (Family member)
	I think at that some care home staff haven’t got a proper insight into what they’re doing so we need proper training that embodies dementia and communication side of things and about care and what to do properly (Care Home staff)
Internal training delivery method	Anything online, it’s all tick-boxing… (Care Home staff)
	I’m not a big believer in online training (Care home staff)
	We deal with people, so we need to learn from each other. We can’t learn from a book, it’s not like we’re learning how to sew or how to build a wall because our… the group that we deal with change on a minute-by-minute basis, second by second. We’ve got to be proactive to react to that and you can’t teach that without experiencing talking to each other. (Care home manager)
	Some [care homes] are very good. Some have just one trainer for everything. One trainer for everything except fire” (Care Home staff)
	More knowledge [is needed] for staff. Stop issuing mickey mouse certificates at £5,000 a throw, and [start] actually training them (Care home manager)
Concerns about legitimacy of Further Education (FE)	The FE system is geared now towards having everybody in and giving them certificates. They turn up saying, “I’m qualified”. Yeah, but what can you do? Well, not much actually (Care Home Manager)
	They were going to scrap Level 2’s in social care… Are you aware of that? The Welsh government were going to scrap it, because they want everyone to have a Level 3. Well, what about the [people] that can’t get a Level 3? It doesn’t matter, you give them the Level 3…. But they’re still issuing the certificate without any training. You can check what I’ve just said, it’s all true” (Care Home Manager)

### Subtheme 1.3: Cognitive Flexibility

Cognitive flexibility as a subtheme revealed relatively even barriers to facilitators (N = 56 barriers and N = 52 facilitators). Here we refer to the core values that staff members bring to the role and how amenable they are to altering thinking patterns in response to changeable situations within the workplace.

The importance of adaptable thinking and the capacity to challenge assumptions was raised by participants. One common discussion was whether people with dementia can change their opinions, perceptions and attitudes. Some participants acknowledged that people with dementia can change and alter in their choices. Others demonstrated views that people with dementia were predictable in their behaviour. Discussion considered people with dementia who may have once preferred a particular diet, but now have a different appetite or taste (Table 8)

**Table 8.** People With Dementia can Change Their Preferences

Some people come into care homes not liking sprouts and when they die, they’re loving sprouts (care home staff)
And, you know, somebody who’s never played bingo in their life and wouldn’t entertain it, suddenly loves it and can’t wait for Wednesday afternoon. And you think, a lot of things change, and they should be allowed to embrace that and enjoy it…. (care home staff)

An example of cognitive flexibility elicited through a vignette that introduced a male resident attempting to get up and go to the toilet during a mealtime (Table 2) identified issues with flexibility to meet individual needs when undertaking other tasks.

Some participants voiced that it was the gentleman’s right to use the toilet whenever he chose and felt he should be supported to do so. Others expressed that although regrettable, it was not the responsibility of the staff in the dining room to assist him as their role at that time was to provide the meals to other residents. The reluctance for some staff to step outside of a prescribed role was discussed in different groups. The attitude of ‘that’s not my job’ (Table 9) was considered a barrier that threatened the autonomy of a person with dementia. Another offered solution was that the gentleman could be ‘trained’ to use the toilet prior to the meal. It was proposed that he likely always asked to use the toilet during the mealtime, and that he and his toileting habit should fit in with the care home routine rather than staff show flexibility towards him. A further suggested solution was announcing to all residents that the mealtime was about to commence and that this was their opportunity to use the toilet. Some participants appeared more aware of the impact on human rights principles that relate to this situation, such as the lack of privacy, which affects the person’s sense of dignity and respect. Violations of the person’s autonomy and choice were recognised in the expectation that the person should adhere to a publicly announced invitation to use the toilet. (Table 9).

**Table 9.** Variances in Cognitive Flexibility

Controlling inconvenient habits	You get people that, um, I’m sure if that’s a resident who’s doing that, they probably do it every time the meal comes… (Care Home staff)
	They’ve got it set in their head at certain times. I’ve even suggested, because I don’t work on that floor, that they take him prior to lunch. But no, he’s got a set clock and that’s his time. It’s usually just as his meal’s served, he’ll have two spoonful’s and then he’s got to go (Care Home staff)
	I would probably say; right, we’re having lunch in ten minutes, does anybody need to go to the toilet… (Care Home staff)
It’s not my role	But I think that it’s not the staff’s problem because they are delivering lunch at that time and that’s their task (Care Home staff)
	Because it’s lunchtime; it’s lunchtime, their focus now is nutrition, got to get everyone to eat, drink, check the… the trolley back in the kitchen, they’ve got to stick to their timescales
	I get, “Oh, it’s not my job” … (Care Home staff)
	That’s a word you hear a lot, isn’t it? ‘It’s not my job.’ (Care Home staff)
A battle to change attitudes	It is a battle sometimes because some carers don’t want to change… it’s not going to happen overnight and you’ve got to chip away at that every day, changing attitudes (Care Home manager)
	But they’ve still got this fixed idea from many years ago where they think, oh, we can’t take him to the toilet at mealtimes (care home staff)
	I’ve got some staff that work in the service, they’ve got a good heart but maybe are still a little bit old in their thinking and it is harder to bring them along with the changes and you’d never put a new member of staff with them because there would be certain traits and things that they may do that…maybe aren’t as politically correct…(Care home manager)

## Theme 2: Organisational Culture

This theme examines the influence of the organisational culture in upholding human rights for people with dementia in care homes. It includes subthemes: **Care home ethos, Organisational flexibility and Staff morale.**

### Subtheme 2.1: Care Home Ethos

This subtheme accounted for the largest number of barriers perceived by participants in relation to upholding principles of human rights. Comparison of barriers to facilitators were N = 29 facilitators as opposed to N = 103 barriers. This subtheme reflects distinctive collective features of the organisation, the moral and ethical standpoints they represent and the principles they demonstrate in connection with the care of people through leadership. Some participants had significant experience of working across different homes within the UK and could compare different practices in relation to leadership, inclusivity, and equality.

Discussion reflected that managers are not always given the decision-making autonomy that reflected their level of accountability and responsibility for the home. Conflict between providers and managers represented a threat to demonstrating a positive care home ethos. One home manager referred to ‘corporate greed’ and alluded to having previously been in a position of subordination by a domineering superior or group within the organisation who did not make decisions according to the needs of the residents. Another manager from a different focus group spoke of a low standard of care and dignity provision in contrast to what the resident was paying to live in the care home (Table 10).

**Table 10.** Corporate Greed

P: Years ago, I left corporate, because of what I called corporate greed.
I: So, when you say corporate, what do you mean?
P: Corporate…one of the big companies, yeah. So, they may have several care homes. You were told what the staffing level would be, and that often didn’t fit with what your residents’ needs were. And I’d get away with it because I was mouthy, and I didn’t care because I put on sufficient staff, I never had agency. I didn’t have any more safeguarding than you would expect. There were lots and lots of people with quite obscure behaviours and needs. So, they were making money out of me, so they left me alone, yeah?
But then when I went and commissioned a brand-new care home, all of a sudden, they wanted me to cut staff, even though these people were paying £1,500 a week for a room. They wanted to cut the kitchen, and I just thought [inaudible] I can’t do this. So now I’m working for people who value staff, and my Nurses are the best paid Nurses that I’ve ever known. There’s not one member of my staff who is under the Living Wage, and we make lots of money. But the residents have huge amounts of staff. So it’s not rocket science…. (Care home manager)
One thing that used to kind of upset me is when I find out how much the residents are paying to be in a specific home. It could be like £4,000 a month. It could be £3,000 pound a month, and to be treated in a way that you’re not cared for to be like kind of, what’s the word, yeah, treated without dignity, to be neglected. That’s like… I feel that’s upsetting and it’s unacceptable, really. (Care home manager)

Care home ethos was also reflected in the inclusivity of people with dementia in both organised activities and general home life (Table 11). The concept of inclusivity reflects all FREDA principles but particularly the values of fairness, equality and autonomy.

**Table 11.** Demonstrating Inclusivity

She even came and sat in on the manager’s meeting and helped herself to the buffet! And nobody… nobody, not even the directors of the company asked her to leave. In fact, they encouraged her, got up, got her up there and she sat by us. She used to come in on all our training….she’d open the door and it was expected of her. We used to pull up a seat for her and she’d sit there quietly (care home staff)

In this example, the management team set the moral standpoint of the home through demonstrating positive and inclusive behaviours towards people with dementia. Conversely, other discussions centred around people with dementia being excluded from activities or outings, hence having implications for the persons fairness, respect, equality and autonomy. There was a propensity towards attending to residents who were easier to engage with, whereas people who exhibited behavioural or emotional needs were often avoided. Some participants conceded that at times people with dementia were not taken on outings due to limited staff numbers or lack of sufficient expertise (Table 12)

**Table 12.** Exclusion of People With Dementia

Sometimes you probably might avoid somebody that you knew was going to be constantly shouting out and repetitive (care home staff)
That’s how we do things, I mean, if it’s something you’re going to do last minute, you’d still be like; oh, we’ve only got this many staff, so this is how many [people] we can manage to take out (care home staff)
They take people that are less difficult. They take the more abled and they end up with somebody that’s less… difficult (care home staff)
P: And it depends also who’s on duty (care home staff)
P: Yeah (care home staff)
P: How much of a rapport they’ve got with them. How good they are (care home staff)
I know that activity coordinators are sometimes a little bit guilty for going to the same residents all the time… (care home staff)
I’d like to think that if I was living in a home, that I was with people that didn’t have dementia as well, and that if I wanted to do something that they were doing that I would be able to do it. And the only consideration that staff would take into account would maybe be my health and safety (Person with dementia)

Participants acknowledged a power inequality between staff and management, and between care assistants and nurses. There were examples of care assistants feeling unable to challenge poor practice of those in more senior positions. This mirrors the autonomy and equality dynamic in relation to staff and residents in some of the examples provided, which subsequently influences a person’s sense of feeling respected and treated with dignity and fairness. Contrary to this, managers who support staff and consider skills of compassion and empathy as assets were far more demonstrative of a positive care home philosophy (Table 13).

**Table 13.** Different Approaches to Leadership

The manager will tell you, “Shut up your mouth, because she’s a nurse”, I don’t care if she’s doing something… You know what I mean? So, just give the power to the nurse, she’s in charge, she can do whatever she wants… (Care assistant)
Having an approachable manager is key, because if you’ve got a manager who enjoys what they do and is giving out that as their ethos, you’re going to have a work force. You know, if you’ve got a manager who shuts themselves way… (Registered Nurse)
One [manager] came in slamming everything around and nobody wanted to be on shift with them, and the other one was really approachable and really nice (care home staff)
I never interview based on their skill level, I interview based on compassion, care…Because you can teach someone a skill but you can’t teach them a feeling (Care home manager)

### Subtheme 2.2: Organisational Flexibility

Organisational flexibility also accounted for a higher number of barriers (N = 80) in contrast with a much smaller (N = 18) number of perceived facilitators. This subtheme reflects the freedoms or constraints of the organisation’s rules, policies, or routines, and how these impact on staff and residents. Discussion contributing to this subtheme related to pressure created by constrictive timescales and staffing shortages (Table 14), which gave way to rigid routines and tasks that impacted on the autonomy and dignity of people with dementia.

**Table 14.** Timescale Pressures

Still got nine o’clock breakfast, 11 o’clock tea, tea trolley, one o’clock lunch, three o’clock whatever, and in-between pad changes and, you know, repositioning, hoisting people or whatever. (care home staff)
The team is like… lots of them are task oriented…. Sometimes that comes in because they have to finish feeding in this one hour, take everything back… everyone back to lounge, so.. (care home staff)
You know, the kitchen then will be saying, ‘You haven’t brought your tea-trolley back, it’s 10 to 12,’ you know. It’s like juggling eggs and you can’t like catch them. (care home staff)
So that was 15 full hoists to all get up. We started at half past seven, to be up in the lounge by half past ten, and that’s including changing them, feeding their breakfast and getting them ready or whatever. It’s just… by half 11, 12 o’clock we [inaudible] you know, not had breaks or anything. (care home staff)
I’ve worked in so many different homes, I’ve come across homes which are mainly time orientated but then there’s homes which are more kind of lenient. So when it comes to breakfast, for example, I know that a lot of homes say, ‘Nine o’clock, on the dot, you need to be down.’ But there are homes which are like… whatever time the resident wants to come down, they’ll bring them down. So it could be until like 11 o’clock, and whoever doesn’t want to come down at nine o’clock can have their breakfast in bed rather than having to bring them down (care home staff)

Participants discussed the organisation’s propensity towards risk aversion regarding people living with dementia. Some homes appeared to be disinclined towards risk more than others. This was spoken about in relation to scrutiny from regulators and others. Good practice was shared regarding being mindful of risks but *managing* those risks carefully rather than *avoiding* them (Table 15).

**Table 15.** Risk Aversion vs Risk Management

Risk aversion	P1: I think we are very risk averse in care homes. We risk assess everybody to the end degree (care home staff)
	P2: Because you’ve got to (care home staff)
	P3: And that’s because of safeguarding (care home staff)
	It’s quite scary for us as well because you’ve got CIW, you’ve got social care world, you’ve got the police behind you all the time so it’s quite scary for staff to… You’ve got to be a specific sort of person to be happy to take those risks, haven’t you? I mean if you work agency, you know you don’t necessarily know the residents inside and out so… so you’ll be even more risk-averse because you’d think… Hang on, I might be in trouble… (Registered Nurse)
	It’s about getting the family on board, management on board, health and safety officer, because like yourself you would have had to do a risk assessment for that
Risk management	I mean we’re working in a very regulated industry… so we have to do it properly. We have to have safeguards in place. But safeguards shouldn’t stop people doing things that they want to do”. (care home staff)
	One of the worst things that we can do to [them], to ostracise them and not allow them to have the same opportunities of someone that hasn’t got dementia. We have to risk assess it carefully, but I think if we’re proactive rather than reactive then it can work, it really can work (Care home manager)
	A couple of times a week, I take my dog into her and she looks after him, in that she’s got a child gate on her room. She’ll stay in the room all day that day. It’s her choice. And she has my dog in with her…. So it’s just a case of risk assessing, and making sure everything’s in place (Care home manager)

The following exchange between a group facilitator and a male participant who is living with dementia in a care home, adds context to this theme, demonstrating the effect of risk aversion on a person. The group had been discussing that some people with dementia were not included in outings due to risk.

Facilitator: Richard (pseudonym), have you ever had any experiences like that?

Richard: Like what, love?

Facilitator: Like everybody going to do an activity and they don’t take you with them?

Richard: Yeah.

Facilitator: Yeah?

Participant: How does that make you feel?

Richard: Lonely

Staff were aware of the activity of CIW, the regulator for care homes in Wales, including unannounced inspections. Likewise, they referred to visits from local health boards and feeling scrutinized by other visiting external specialists. Many comments made in connection with regulators viewed them as coming from a place of poor understanding, having unnecessarily high expectations, punitive, and inconsistent in their approach (Table 16).

**Table 16.** External Scrutiny

P1: I think, you know, you’ve got your regulators, you’ve got the government or whatever, sitting in offices never setting sight on a care home, only to walk in and say, “Huh, what’s that doing there? Huh, why is that there? (Care Home staff)
P2: So, they’re coming in with a negative point of view straight away… (Care Home staff)
Yeah. And, um, you know, rather than actually stopping and looking and spending time, you know, that they might walk in and see somebody slumped over the corner of the arm of the chair and go away and report you for that, right? (care home staff)
I agree with you totally about, you know, how… how much we’re regulated, because it’s not just CIW and [Health Board], the fire people and the environment health people and everybody else who wants to come through the door… (Care Home manager)
I work in some care homes, when you’ve got a care home that’s really terrible, but the report shows ‘outstanding’. Or you could get a bad report and be a good home. (Care Home staff)
I was at a Social Care Wales meeting and this woman was talking about the qualification changes and I said, has anybody in your offices ever worked in a care home? And she refused to answer me! (care home manager)

The perceived scrutiny from external regulators appeared to contribute to organisations’ restrictions and their reticence to be more flexible. Concerns about inspections and potential safeguarding reports were mentioned often.

### Subtheme 2.3: Staff Morale

In this subtheme, coded comments comparing barriers to facilitators were N = 43 (barriers) as opposed to N = 4 (facilitators). Some care home staff discussed feeling burnt out and undervalued (Table 17). Various influencing factors have already been discussed, including unsupportive leadership, regulatory scrutiny and timescale pressure. Participants reflected on the impact of public perceptions on care homes, as well as the lack of career pathway for potential new recruits. There were limited comments reflecting factors that increase staff morale. However, some participants vocalised the importance of showing appreciation to staff and recognising humanity and equality, which also link back to the theme *emotional intelligence*.

**Table 17.** Staff Morale

Burnt out	But then you get people… who get to the stage where they… they don’t want to come into work so they just ring and they use any excuse, ‘Oh, I’m not coming in today because I’m…’ whatever, because their heart’s not in it, because they know that they’re going to be there for 12 and a half hours or whatever, and they’re going to come out and they’re going to be absolutely, you know… so that’s the burn out (care home staff)
	Now, I’m virtually a zero-hour contract firm. You know, so if I’m off on the sick it’s statutory sick pay, or whatever. So, your staff… because, you know, you do feel… you just get burnt out… (care assistant)
Not valued	Because that’s what I’ve heard going to a lot of homes, is that… the staff always saying, ‘Oh, we’re not appreciated.’ Why am I [inaudible] myself out, basically, but not being thanked, or… yeah, I’m not feeling valued for what I do. (care home staff)
Other’s perceptions	It’s the same with care homes, isn’t it? Because we only ever hear about the bad things…. we never hear about the good things. (care home staff)
	I was a sister in [name of hospital omitted for confidentiality] and then I had to stop [and worked in] a care home…. people say oh you are a retired nurse now and it makes me really angry; there are so many nursing skills that I am using every day (care home staff)
	P1: We’ve been getting fed up as carers of the sort of the media… and them focusing on the really bad, bad care homes...when we’ve got lovely care homes and nobody wants to know about, it’s just a shame.
	P2: Unfortunately, good practice doesn’t make news (care home staff)
Unpopular choice for students	When I found out that I had a nursing home placement, and I was telling people they were going “Awwwww you poor thing!” (Student nurse)
Lack of career pathway	[We should] try to fight to get nurse practitioners in care homes to give that structure of a where nurses could go for a career pathway. You don’t want to be stuck as a band five in a nursing home for the rest of your life (Care home manager)
Positive contributions to morale	I always go around at the end of the shift saying, thank you guys for today, because they don’t get appreciated for what they do. (Care home manager)
	The term I hate is, ‘I’m just a.’ There no such thing as, ‘Just a,’ whether it’s just a carer, just a cleaner, just a ‘whatever’. There’s no such thing. We are all the same level (Registered Nurse)

In the above, staff reflected on situations that reflected infringements of human rights values within their daily work. The sense of dignity and respect, fairness and autonomy encountered within their role were absent in these reflections. Hence there was the inference that it is difficult to provide value to others when one does not feel valued themself.

## Theme 3: External Influences

There were two main outside contributors that directly influenced a care worker’s ability to uphold human rights principles for people living with dementia in care homes. These were **social care funding and support** and **family and visitors**.

### Subtheme 3.1: Social Care Funding and Support

This subtheme accounted for the smallest number of coded comments reflecting facilitators (N = 1) in contrast with barriers (N = 35). Participants referred to poor external funding which influenced staff pay, and budgetary restrictions that impact staffing levels and provision of care for residents. Other comments alluded to a sense of care homes being isolated and misunderstood by external professionals whose support would be helpful. This includes interdisciplinary colleagues perceived as either lacking in education about dementia or in possession of a poor attitude to care homes generally (Table, 18). Although the subtheme *staff morale* has been discussed (Table, 17), it is important to note that the following would also impact on how staff feel about their role.

**Table 18.** Social Care Funding & Support

Poor funding and pay	The Government is asking our workforce to be highly educated, highly skilled but paid peanuts… (Care home manager)
	You could get more sitting on a till in Tesco (care home staff)
	We are underfunded, we’re never going to have enough staff, the care… the care sector gets forgotten (Care home worker)
	And then you ask somebody who’s on £8 an hour to be on a register. Really? I’d rather work in Tesco then (care home staff)
	I don’t think that they… the government put enough funding into it for us to be able to attract the right sort of people (care home staff)
	As managers it’s so stressful to try and balance person centred care, staffing levels, staff education, staff morale, staff mental health wellbeing on a 50 pence pot” (Care home manager)
	There’s just not enough funding to give everybody the education they need (care home staff)
	Are you aware that in [name of care home provider omitted for confidentiality], the only sector in the whole bloody world, if you’re successful they reduce your fees! If you’re doing your job well, they give you less money – how does that work? (Care home manager)
Lack of external support	Social workers are now playing the blame game. Because we have had two people fall, now unless I physically sat on that person to keep them in a chair there’s absolutely no way I was going to stop that person getting up because she wanted to walk around… (care home staff)
	I think that starts with… some of the GPs don’t know enough (care home staff)
	I think one of the answers is to stop treating care facilities as warehouses (Person with dementia)
	District nurses who think it’s acceptable to do injections in the lounge because, my gosh, we can’t spend 10 minutes… to transfer the lady or gentleman to the clinic or the nice big room they can use so they actually feel like they’re visiting a nurse (care home staff)
	I work in the hospital as well and they say that dementia care they’ve got all the training, but I don’t think they – they haven’t got it. They haven’t got a clue about how to deal with the people who just you know they need someone to monitor the hospital staff. They need more education (care home staff)

### Subtheme 3.2: Family and Visitors

The presence of friends and family of people with dementia were perceived as both barriers and facilitators by care home participants (N = 37 barriers as opposed to N = 24 facilitators). Enabling factors regarding families included good communication, supportive inclusion, learning about residents through observing family interactions, and joint education opportunities. Impeding factors consisted of perceptions that families were risk averse, overprotective, and unwilling to accept that the person with dementia has changed. The latter reflected that a perceived limited cognitive flexibility in families was contributory. There were perceptions of jealousy from family members towards their loved one’s relationship with care home staff as well as conflicting beliefs regarding approach to care. At times care home staff felt protective of the resident as they did not feel the family were as understanding of their needs as they were and this lack of understanding impacted on the person with dementia’s values of autonomy and fairness (Table, 19).

**Table 19.** Family Influences

Family who are risk averse	But sometimes it’s the relatives as well, they don’t want their families to go out to fall, to get dirty you know my mum is here for you to for you to look after her and keep her safe (Care Home staff)
Opposing views on approach to the person	Some of the families comes and calls a resident by their name than Mum or Dad…
	Sometimes, I’ll… I feel like, oh, is that right or wrong? (Care Home staff)
Conflict regarding the relationship between resident and staff	Sometimes the families get a bit jealous (Care Home staff)
	Sometimes it felt that the staff there knew her more than you ever did (Family member)
Family unable to accept a person has changed	There is a man who supposed to be a vegetarian right, and when you come round with the meal choices, he says “ooh I will have beef pie!”
	So, then his partner will come in and say why is he eating that? Well, that was his choice for lunch like. Because his tastes change. He had chicken pie yesterday (care home staff)
	This friend lived abroad, and when she last saw the resident, she loved a cup of tea with two sugars. She liked two biscuits. She doesn’t anymore. She likes coffee, with one sugar, and she likes a piece of cake. She won’t eat a biscuit. And I explained it and she said, ‘No, no. She likes a cup of tea.’ So, in the end…
	I turned to the resident, and I said, ‘What would you like?’ Because she didn’t even ask her. I said, ‘What would you like to drink?’ She went, ‘Cup of coffee, one sugar and a nice slice of cake.’ And I just went and made it for her. I thought, ‘No, you can’t presume. You can’t presume that they’re the same (care home staff)
Family not understanding of dementia	A lot of the challenges relatives have is through lack of knowledge. They don’t understand why certain behaviours are displayed. They don’t understand some of our reactions to those behaviours, and it’s down to them never having been informed of what dementia is.. (Care Home staff)
	All sitting lovely having Christmas lunch and the family come in and they sit there, and they sit there, would you like some… no, I don’t want anything to eat because I’m going home for my dinner, mum’s dinner’s here and they just stare at her, so what does mum do… She don’t eat her dinner then, does she! (Care home staff)
Family demanding	But sometimes they say, “Can you move that person who is making noise?” (care home staff)
	Some families are a bit over-bearing, aren’t they? (care home staff)
Good communication with the family- an enabler	For me as a relative A lot of it would be gaining a deeper insight and understanding into these issues. Because today has been quite education for me and listening to you now, I’m starting to learn more and more which is really helpful (Family member)
	It’s usually the family that you meet first and if you don’t get the family on side then almost, you’re already on a loser, aren’t you? (Care home staff)
	Again, so we’re all saying, work with the family (care home staff)
	I think we’ve had the practice in one of the nursing homes where I’m working to train the family, actually” (care home staff)
	I just think the carers need to focus more on the social care rather than just [the resident’s] health. You know, families come in to see them, they don’t want to know how many times people have gone to the toilet every day. But they want to know what they’ve done, are they happy, are they being involved? (Family member)

Family members who attended as participants were able to share their experiences of feeling unsure about their place in the home, and the changed relationship with their loved-one. One family member acknowledged the close relationship between their parent and the staff, sharing that they felt the staff knew them better. Communication within the focus group between staff and families was acknowledged as a positive learning experience.

## Discussion

The FREDA (Curtice & Exworthy, 2010) principles of human rights include fairness, respect, equality, dignity and autonomy. Legislation (Equality Act, 2010; Human Rights Act, 1998; Mental Capacity Act, 2005) stipulates these values must be upheld by law. Globally, the Office of the United Nations High Commissioner for Human Rights (OHCHR, 2025) and WHO (2017b) strongly advocate for the human rights of people living with dementia. Likewise, third sector organisations (Alzheimer’s Society, 2021; Alzheimer’s Disease International, 2024; British Institute of Human Rights, 2021; Dementia UK, 2023) as well as other human rights initiatives (John’s Campaign, 2014, A Person Like Me, 2019) campaign for people living with dementia to be treated with dignity, respect, fairness and advocate that they have the right to autonomy and choice.

Previous research that considered the application of a human rights approach to people living with dementia in care homes (Kinderman et al., 2018) found no significant enhancement in the quality of care received. Further research was recommended to identify staff attitudes that translate to behavioural outcomes. This research has addressed many of these factors by highlighting the contributory influences of emotional intelligence, cognitive flexibility and care home ethos. Other outcomes of this study are prevalent within the existing literature. These include the influence of organisational culture in care homes (Killett et al., 2016), problems associated with staff morale (McKenzie et al., 2016) and burnout (White et al., 2020). Relationships between care home staff and families have been studied previously (Hoek et al., 2021). The requirement for improved educational provision (Rooney, 2019) and social care funding are likewise not new concepts. However, this study’s findings being analogous with earlier published work not only reinforces the findings of previous research but confirms that these factors remain present in the care home setting.

### What This Research Adds

This is the first study to elicit the opinions of heterogenous groups of key stakeholders in ascertaining the enablers and constraints to supporting human rights values for people with dementia who reside in care homes. This research is also the first to determine that emotional intelligence and cognitive flexibility are key influences in enabling staff to uphold values human rights for people with dementia in care homes. This is encouraging, since both emotional intelligence and cognitive flexibility are variables that can either be modified through appropriate education (Serrat, 2017, Lee et al., 2024), modelled by leaders as influencers of care home ethos, or purposefully sought via selective recruitment (Mansel & Einion, 2019; Khassawneh et al., 2022).

### Emotional Intelligence and Cognitive Flexibility

The seminal work of Goleman (1995), describes emotional intelligence as the ability to recognise, understand and navigate emotions. It encompasses self-awareness, self-regulation, social skills, empathy, and motivation (Goleman, 2020, Khademi et al., 2021). Being emotionally intelligent involves honesty, authenticity, genuineness, and the ability to embrace ones’ own vulnerability (Sheard & Butcher, 2022). Self-awareness is an important factor; being mindful of ones’ own thoughts, attitudes and actions through self-reflection is essential (Clifford & Doody, 2018). This involves the practice of self-compassion (Abdollahi et al., 2021; Clouston, 2021). Closely linked to emotional intelligence is the concept of *cognitive flexibility*. This refers to the ability to swiftly modify and reorganise ones’ thinking patterns to shift between different tasks (Kruczek et al., 2020). For healthcare staff it would mean altering ones’ mindset, adjusting priorities and adapting to continuous changes within the working day (Dehghani & Bahari, 2021). It is a cognitive process which allows a person to navigate their own internal aims alongside the present reality (Braem & Egner, 2018).

Although two different processes, emotional intelligence and cognitive flexibility share similarities in that both require a willingness and ability to be flexible and adapt one’s usual approach (Wu et al., 2021). Both require self-awareness. In the case of emotional intelligence, it is essential to be aware of one’s own attitudes, thoughts and emotions (Khademi, Abdi, Saeidi, Piri, & Mohammadian, 2021), while simultaneously considering the emotional state of others (Goleman, 2020) and how our actions might impact on another individual. Similarly, cognitive flexibility requires the ability to accommodate different viewpoints, weigh up different options and adapt to changes (Wu et al., 2021). These intersecting characteristics were both important themes in relation to enhancing staff’s ability to support human rights. However, emotional intelligence was the most prominent facilitator. The author’s surmise that this is because caring for people with dementia requires emotional labour (Funk et al., 2021) and staff can experience moral distress in their working role (Kim et al., 2025) which brings the emotional aspects of caring for someone with dementia to the fore.

### Emotional Intelligence and Cognitive Flexibility Demonstrated by Care Home Participants

Participants of this study exhibited varying levels of engagement with the emotional and cognitive aspects of working with people living with dementia. In the ‘therapeutic lie’ example, willingness to consider the grey area that exists between the concrete ‘yes or no’ response, expresses both emotional intelligence and cognitive flexibility in action. Some participants recognised the delicate intersectional balance between employing compassionate deception (Skov et al., 2024) and maintaining a trusting relationship with the person living with dementia. There was acknowledgment that deliberately misleading someone with dementia is incongruous with human rights principles of dignity and respect, in addition to professional codes of conduct and personal morality (Villar & Martínez, 2024). However, there was recognition that in many cases the alternative ‘blatant truth telling’ would most likely cause the person distress. Navigating this ethical dissonance requires both emotional intelligence and cognitive flexibility, as it requires simultaneous emotional engagement in addition to the cognitive knowledge to choose a thoughtful response. This was demonstrated with thoughtful fluidity by some participants, whereas others offered more concrete responses to these important ethical considerations. The diverse responses reflect a likelihood that people with dementia will receive innumerable approaches to their emotional care and wellbeing in practice. This is of concern, as people with dementia require an approach that is consistent with *their* unique emotional and cognitive state, not one that reflects the caregiver’s preference.

The gradient of responses suggests that there are varying levels of emotional intelligence and cognitive flexibility in care home staff. Receiving diverse responses in relation to questioning ‘Where is my husband?’ (who is long deceased) for example, is likely to lead to increased confusion, anxiety and contribute to mistrust of caregivers. Likewise, ignoring the question and simply using distraction techniques leaves the person feeling misunderstood and with unmet needs (Wolverson et al., 2021). It is important for staff to understand that often such questions are an expression of unfulfilled emotional needs such as loneliness or a need for social interaction (Cohen-Mansfield et al., 2015). Similarly, they may be seeking the feeling of security that their loved one had once provided them (Alzheimer’s Society, 2023). As people with dementia can experience different levels of awareness at the same time (Feil, 2017) it is important to understand the emotional context and validate the person’s feelings, rather than provide a perfunctory response that involves either a form of remedial truth or reality orientation (Oliveira & Sousa, 2021). The importance of empathy and validation are well-documented in seminal works (Feil & Altman, 2004; Neal et al., 2003). Understanding the emotions of a person who may be experiencing fluctuating realities can be complex and achieving success with this involves engagement with both thoughts and emotions with fluidity.

### Emotional Intelligence: Inherent or Teachable?

Participant comments reflected a belief that some of the fundamental elements of emotional intelligence, such as empathy and compassion, are inherent within a person and therefore cannot be taught. However, in contrast to character or temperaments, empathy is considered a skill that can be “invoked, taught, re-awakened, and nurtured” (Thangarasu et al., 2021, p. 2). Mattingly and Kraiger (2019) meta-analysis of studies involving emotional intelligence training in adults demonstrated a moderate positive influence on emotional intelligence irrespective of study design.

Serrat (2017) contend that emotional intelligence can be learned, but requires personal motivation, practice, and the willingness to accept feedback. It is recommended that dementia education should encompass the importance of emotional intelligence (Rooney, 2019; Teskereci et al., 2020). This has been shown to improve the care of residents and enhance staff wellbeing and empowerment (Karimi et al., 2020). Likewise, emotional intelligence education is considered to improve relationships and work performance (Foster & McCloughen, 2020; Kotsou et al., 2019) and enhance caring behaviours of nurses (Nightingale et al., 2018). It has also been shown to increase job satisfaction, which can reduce staff turnover (Laing-Hall, 2023). This ultimately can reduce levels of burnout (Soto-Rubio et al., 2020) experienced by staff. Likewise, higher levels of cognitive flexibility correlate with improvements in nurse’s attitudes towards professional autonomy (Kılıç et al., 2024), promote coping skills and psychological adjustment (Kruczek et al., 2020) and helps alleviate the psychological impacts associated with traumatic events experienced by nurses (Sarpdağı et al., 2025). There is evidence to suggest that cognitive flexibility can be taught successfully to healthcare students through various approaches such as transactional analysis training, (Abbasszade et al., 2025), psychodrama-based interventions (Çataldaş et al., 2024) and cognitive training (Lee et al., 2024).

### Cognitive Flexibility and Emotional Intelligence Jointly Influence Organisational Culture

Findings of this study considered that *care home ethos* and *organisational flexibility* were the greatest perceived barriers in relation to supporting human rights for people with dementia. These were part of the subtheme *organisational culture*. As noted, this has been reported in the literature previously. However, there are important associations between *emotional intelligence*, *cognitive flexibility* and their impact on *organisational culture.* Here we discuss how each of these important subthemes impact on one another.

Although cognitive flexibility and emotional intelligence are different concepts, they are connected in relation to how staff respond to certain situations that conflict with their internal expectations or goal at that time. One example was seen in the discussion concerning the gentleman requiring assistance to the toilet during a mealtime. Those who lack *cognitive flexibility* may be fixed in a view of what their role was at that time; to assist with the meal. The person requiring assistance may be denied this aspect of personal care if staff are unable to consider alternatives or switch their view of ‘the task at hand’. This may result in the gentleman becoming agitated, possibly soiling himself publicly, and lead to understandable emotional disturbance. The suggestion of announcing to all residents that the mealtime was about to commence and that this was their opportunity to use the toilet, demonstrates an attempt to control the potential for further disruption by introducing a new unofficial ‘rule’. With this would come the unspoken tenet that it was unacceptable to use the toilet during a meal. Hence, this is incompatible with ‘autonomy’ as a human rights value.

Brooker and Latham (2015) describe how if repeatedly unacceptable ways of responding to a person with dementia go unchallenged, they can quickly become normalised within the *organisational culture*. Attitudes and actions that disempower, ignore, infantilise or disparage a person was described originally in the seminal work of Kitwood (1997) as ‘malignant social psychology (MSP)’. Although seldom considered malicious in original intent (Brooker & Latham, 2015), controlling behaviours can be replicated by other staff and soon be deemed an ‘acceptable’ part of care delivery. Caregivers who can contemplate different solutions to competing demands by temporarily shifting the focus of their present task, would be more likely to uphold the persons’ dignity (Mehralian et al., 2024).

Reflecting on the current scenario further, the importance of the gentleman’s predicament must first be recognised and be considered a priority, prior to someone being able to exercise cognitive flexibility. If perceived as simply ‘yet another task’, it would not factor as important to warrant flexibility. Without the *emotional intelligence* to empathise, the gentleman’s distressed behaviours may simply be construed as ‘challenging’ (Cohen-Mansfield et al., 2015; Daly et al., 2015) rather than an expression of physical or emotional need (Petty et al., 2018). Staff may view the person’s anguished response only in the context of their dementia diagnosis; he has dementia and that is why he is agitated. This is a form of diagnostic overshadowing (Pepper & Dening, 2024) that can lead to under recognition of important physical and emotional health needs. In attributing liability for the agitation to the diagnosis of dementia, the caregiver might feel released from the expectation to think critically, empathise or act outside of their prescribed role. This disassociation can be a response to burnout.

Considering the results of this study alongside evidence from the existing theory, Emotional intelligence and Cognitive flexibility are both important requirements to delivering care that supports human-rights values for people living with dementia.

### Limitations and Future Research Directions

There is known disparity between care home services (Killett et al., 2016; CQC, 2021). Hence, it cannot be assumed that participant comments are representative of the attitudes and understandings of staff in care homes either across Wales, nationally or globally. Given the context of a civic mission stakeholder event about empathy and human rights, care home participants who responded to the invitations may represent homes that are well-led and who already embrace human rights values for people living with dementia. Likewise, participants who were in senior positions may not exemplify the approach of all who work in the care homes represented. Further, they may be reluctant to share their challenges or shortcomings (Bergen & Labonté, 2020) and present only good practice. However, researchers mitigated this by facilitating a supportive and open atmosphere, with the reassurance of confidentiality. A further limitation is that due to the heterogenous group, when reading the transcriptions and listening to the recorded discussions it was sometimes difficult to ascertain the specific roles of the care home staff who were speaking. Therefore, it was not always possible to distinguish between a comment made by a Registered Nurse, Care Assistant or Activities Co-ordinator. However, comments made from the smaller number of people with dementia, family members and student nurses were more readily apparent. In situations where roles were easily identified, their title (of ‘Registered nurse’ or ‘Care home manager’) was attributed to the specific comment. However, if the role was not distinguishable, they were referred to as the more generic term ‘Care home staff’ next to their comment.

This research has implications for directing the approach to care home staff recruitment, the focus of education within care homes and guidance for care home managers on flexible, emotionally intelligent leadership and role-modelling. It may also be utilized in higher education institutions to support curriculum development to include greater emphasis on emotional intelligence and cognitive flexibility in healthcare education. Furthermore, it may influence policy, to establish a greater insight into the lived experience, which may enable the development of strategies that will more accurately address service gaps and initiatives to support those affected by dementia. The findings of this study have the potential for broader applications for the care of other vulnerable patient populations, such as those who live with mental health conditions, disability, or those with intersectional attributes that may place them at risk of human rights violations. It is relevant for not only care home settings, but acute care facilities, domiciliary care, or outpatient departments. Additionally, it would be valuable education for developing countries to help support new approaches to healthcare. Future research should aim to identify the specific aspects of emotional intelligence that are likely to enable staff to uphold human rights for people with dementia and consider more specifically what approach to educating staff will more reliably enhance cognitive flexibility. This would inform the content and approach to education, supervision and support for staff who work in care homes.

## Conclusion

People with dementia are at risk of experiencing inequalities and other contraventions of their human rights. This qualitative study explored the experiences of key stakeholders in understanding the factors that enable or restrict the facilitation of human rights principles for people living with dementia in care homes. Researchers facilitated focus groups of heterogenous stakeholders to elicit the understandings in relation to upholding these principles.

Researchers drew on the Braun and Clarke (2006, 2019, Braun et al. (2022); Braun & Clarke (2024a, 2024b)) reflexive thematic analysis method to scrutinise, understand, interpret and report the data from transcriptions and audio recordings. Personal attributes of *emotional intelligence* and *cognitive flexibility* were the most important influences on the ability of staff in care homes to support human rights for people with dementia. This adds a fresh perspective to this field of study and has implications for the direction of future care delivery in care homes.

This research is helpful in adding to an existing body of knowledge in relation to human rights-based approaches to caring for people living with dementia. It also offers new thinking and direction in the support of people without dementia living in care homes, as well as having relevance to the care of people with dementia in hospitals and community settings.

## Data Availability

The researchers took precautions during data collection to protect the identity of focus group members by offering the option of participants providing a first name only for their name badge. Additionally, no care home names were included on name badges or lists that were viewable to other stakeholders. Care home participants were reassured that the names of their homes would not be identified. Despite this, names of care homes and participants were inadvertently mentioned by participants during focus groups. Some include very sensitive information. Therefore, unfortunately it would not be possible to share the research data in its entirety (to include transcriptions or recordings) in a public data repository.
